# A nomogram for predicting the risk of venous thromboembolism in patients with solid cancers

**DOI:** 10.1007/s11239-023-02856-0

**Published:** 2023-07-18

**Authors:** Siyu Chen, Wei Sun, Min Dan, Yue Sun, Yongsheng Jiang

**Affiliations:** Department of Oncology, Tongji Hospital, Tongji Medical College, Huazhong University of Science and Technology, 1095 Jiefang Avenue, Wuhan, 430030 Hubei China

**Keywords:** Venous thrombosis, Solid cancer, Prediction model, Nomogram

## Abstract

Cancer patients with venous thromboembolism (VTE) are prone to poor prognoses. Thus, we aimed to develop a nomogram to predict the risk of VTE in these patients. We retrospectively analyzed 791 patients diagnosed with solid tumors between January 2017 and May 2021 at Tongji Hospital. Univariate logistic analysis and multivariate logistic regression were adopted in this study. Our results indicated that age ≥ 60 years, tumor stages III-IV, platelet distribution width (PDW) ≤ 12.6%, albumin concentration ≤ 38.8 g/L, lactate dehydrogenase (LDH) concentration ≥ 198 U/L, D-dimer concentration ≥ 1.72 µg/mL, blood hemoglobin concentration ≤ 100 g/dL or the use of erythropoiesis-stimulating agents and cancer types were independent risk factors. The nomogram prediction model was developed based on the regression coefficients of these variables. We assessed the performance of the nomogram by calibration plot and the area under the receiver operating characteristic curve and compared it with the Khorana score. The concordance index (C- index) of the nomogram was 0.852 [95% confidence interval (CI) 0.823 to 0.880], while the Khorana score was 0.681 (95% CI 0.639 to 0.723). Given its performance, this nomogram could be used to select cancer patients at high risk for VTE and guide thromboprophylaxis treatment in clinical practice, provided it is validated in an external cohort.

## Title Page Abstract

- Compared with the general population, patients with tumors are four to seven times more likely to develop venous thrombotic events.
- Thromboprophylaxis treatment increases the risk of bleeding complications and the risk of VTE varies widely among individuals.
- Only patients at high risk of thrombosis will benefit from primary thromboprophylaxis.
- This intuitive nomogram model with variables available in routine clinical practice to quantify the risk of VTE in patients with solid cancers and assist clinicians in tailoring anticoagulant therapy.

## Introduction

Venous thromboembolism (VTE) is a prevalent complication in cancer patients [1]. Compared with the general population, patients with tumors are four to seven times more likely to develop venous thrombotic events [2–4]. A population-based cohort study showed that the incidence of venous thromboembolism in these patients has increased steadily over the past decade [5]. Cancer-associated thrombosis may result in discontinuation of antineoplastic therapy, decreased quality of life, and increased mortality [6]. Clinical trials indicate that prophylactic anticoagulation leads to a significantly lower rate of thrombotic disorders [7, 8]. However, because thromboprophylaxis treatment increases the risk of bleeding complications and the risk of VTE varies widely among individuals, only patients at high risk of thrombosis will benefit from primary thromboprophylaxis. In this setting, the use of anticoagulant drugs is challenging.

Several prediction models have been created to identify tumor patients at high risk of VTE. The most commonly used and extensively validated score is the Khorana score, which consists of five items: site of cancer, body mass index (BMI), platelet count, leukocyte count, and hemoglobin level [9]. Based on the total points, patients are divided into low-risk, intermediate-risk, and high-risk groups. However, a large number of external validation studies have reported inconsistent conclusions about this model. A systematic review and meta-analysis indicated that the performance of the Khorana Score is not ideal; it has poor prediction accuracy for patients with lung cancer and hematologic malignancies; and more importantly, only 23.4% of thromboembolic events occur in patients assessed as a high-risk group by this model [10]. Other risk assessment models, with modest applicability in routine clinical practice, need further improvements [11–13]. Currently, an accurate clinical prediction model for predicting the risk of VTE in patients is urgently needed.

Previous studies have identified various risk factors for cancer-associated thrombosis, including patient-related factors, cancer-related factors, and laboratory biomarkers [14, 15]. In this study, we attempted to develop an intuitive nomogram model with variables available in routine clinical practice to quantify the risk of VTE in patients with solid cancers and assist clinicians in tailoring anticoagulant therapy.

## Materials and methods

### Study population

**Fig. 1:**
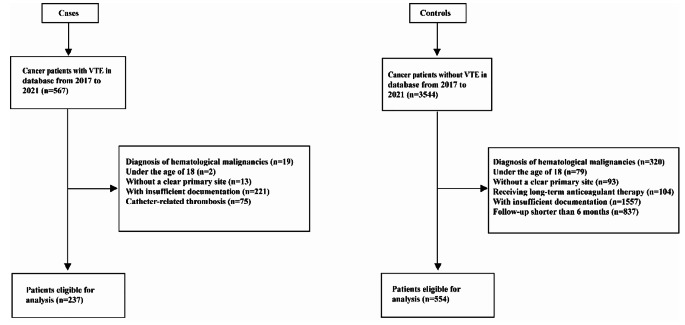
A flowchart to present the inclusion and exclusion processes of this study. VTE, Venous thromboembolism

A total of 4111 cancer patients were identified from Tongji Hospital between January 2017 and May 2021. The inclusion criteria were (a) a histological diagnosis of solid cancer and (b) age older than 18 years. The exclusion criteria included the following: (a) received long-term anticoagulant therapy; (b) did not have a clear primary site of malignancy; and (c) had a follow-up shorter than 6 months. Finally, 791 patients were included into analysis. The inclusion and exclusion processes of this study are presented in a flowchart (Fig. 1). This retrospective case-control study was approved by the Ethics Committee of Tongji Hospital, Tongji Medical College, Huazhong University of Science and Technology.

### Data collection

Data regarding relevant variables were collected according to previous studies and clinical judgment. These variables included age, gender, body mass index (BMI), the primary site of the tumor, tumor stage, personal history of VTE, use of erythropoiesis-stimulating agents, comorbidities including infection, renal impairment, liver impairment, pulmonary disease, diabetes mellitus, cerebrovascular disease, varicose veins, coronary artery disease, hypertension, hyperlipidemia, atrial fibrillation, and prechemotherapy laboratory variables, including platelet count, leucocyte count, hemoglobin concentration, mean platelet volume (MPV), platelet distribution width (PDW), albumin, lactate dehydrogenase (LDH), D-dimer, and fibrinogen. The BMI values and pre-chemotherapy laboratory variables were tested and collected within one week before the first cycle of chemotherapy. And the tumors were divided into stages I-II and stages III-IV according to the AJCC eighth edition TNM-staging system. The defined outcomes were symptomatic VTE, including deep vein thrombosis and pulmonary embolism. The thromboembolic events were confirmed by imaging methods, including venous ultrasound, and computed tomography pulmonary angiography.

### Statistical methods

In this study, 791 medical records were included into the final analysis. Considering the rule of ten events per variable (EPV) and that the occurrence rate of VTE in cancer patients is 5–20%, the amount of data was considered sufficient to create a model [16]. In a few numeric variables, some information was missing. BMI had the most missing values (n = 123, 15.5%); the other variables had very few (< 5%). These missing data were filled by the mean of the group with the same outcome. T-test or Mann-Whitney U test was performed for the analysis of continuous variables. The Chi-square test or Fisher exact method was performed for the analysis of categorical variables. Considering that the ranges of some continuous variables were wide, we turned the continuous variables into categorical variables by the cut-offs to make the model easier to use. Univariate logistic analysis was used to identify the risk factors for VTE. The variables with P < 0.05 were included in a multivariate logistic regression model to screen the independent risk factors. A nomogram prediction model was developed based on the regression coefficients of the final variables. The area under the receiver operating characteristic (ROC) curve and calibration plot were used to evaluate the discriminative and calibrating abilities of the nomogram. The bootstrap method with 1000 replicates was applied to internally validate the nomogram. Finally, decision curve analysis (DCA) and integrated discrimination improvement (IDI) were used to assess the clinical benefit of the nomogram compared with the Khorana risk score. All statistical analyses were performed using SPSS version 23.0 and R software version 4.1.2.

## Result

### Patient characteristics

Finally, we collected the medical records of 237 cancer patients with VTE and 554 without VTE. The characteristics of the patients enrolled in this study are displayed in Table 1. In total, 213 patients were diagnosed with deep vein thrombosis, 23 patients with pulmonary embolism, and 1 patient with both. The time period between cancer diagnosis and VTE diagnosis varied from 1 month to 24 months (median, 1 month). The VTE group and non-VTE group were comparable in terms of gender, BMI, and most comorbidities (P > 0.05). According to previous studies and our findings, cancer type was grouped as very high risk, high risk, and low risk. Pancreatic cancer was divided into the very high risk group (n = 8); bladder, brain, kidney, stomach, esophagus, lung, liver, colorectal, prostate, uterus, and ovary cancers the high risk group (n = 627); breast, oral cavity, and laryngeal cancers the low risk group (n = 156).

**Table 1:** Demographic and clinical characteristics of the study population

Variables	No-VTE(n = 554)	VTE(n = 237)	P value
Age(years), (mean, SD)	54 (10.7)	61 (10.2)	< 0.001
Gender, (n, %)			1.000
Female	285 (51.4)	122 (51.5)	
Male	269 (48.6)	115 (48.5)	
BMI (kg/m^2^), (mean, SD)	22.6 (3.0)	22.2 (2.9)	0.171
Cancer type, (n, %)			< 0.001
Pancreas	3 (0.5)	5 (2.1)	
Stomach	81 (14.6)	22 (9.3)	
Colorectal	116 (20.9)	38 (16.0)	
Esophagus	20 (3.6)	6 (2.5)	
Liver	20 (3.6)	12 (5.1)	
Lung	108 (19.5)	77 (32.5)	
Brain	6 (1.1)	7 (3.0)	
Bladder	3 (0.5)	6 (2.5)	
Breast	96 (17.3)	9 (3.8)	
Gynecologic	52 (9.4)	34 (14.3)	
Nasopharynx	19 (3.4)	2 (0.8)	
Kidney	3 (0.5)	7 (3.0)	
Prostate	4 (0.7)	4 (1.7)	
Others	23 (4.2)	8 (3.4)	
Tumor stage ,(n, %)			< 0.001
I-II	212 (38.3)	33 (13.9)	
III-IV	342 (61.7)	204 (86.1)	
**Laboratory variables**			
Platelet count ≥ 350 × 10^9/L, (n, %)	65 (11.7)	29 (12.2)	0.936
Leucocyte count ≥ 11 × 10^9/L, (n, %)	28 (5.1)	30 (12.7)	< 0.001
Heamoglobin ≤ 100 g/dL or using erythropoiesis-stimulating agents, (n, %)	47 (8.5)	63 (26.6)	< 0.001
MPV (fL), (mean, SD)	10.9 (1.2)	10.6 (1.1)	< 0.001
PDW (%), (mean, SD)	13.0 (2.7)	12.3 (2.5)	< 0.001
Albumin(g/L), (mean, SD)	41.1 (4.4)	37.6 (5.1)	< 0.001
LDH(U/L), (mean, SD)	195 (133.4)	247 (165.1)	< 0.001
D-Dimer (µg/mL), (mean, SD)	1.45 (2.12)	4.91 (5.66)	< 0.001
Fibrinogen (g/L), (mean, SD)	3.72 (1.17)	3.96 (1.34)	0.012
**Comorbidities** (n, %)			
Personal history of VTE	1 (0.2)	4 (1.7)	0.050
Infection	2 (0.4)	0 (0.0)	0.878
Renal impairment	0 (0.0)	1 (0.4)	0.662
Liver impairment	12 (2.2)	8 (3.4)	0.456
Pulmonary disease	7 (1.3)	6 (2.5)	0.327
Diabetes mellitus	39 (7.0)	21 (8.9)	0.460
Cerebrovascular disease	9 (1.6)	10 (4.2)	0.054
Varicose veins	0 (0.0)	1 (0.4)	0.662
Coronary artery disease	3 (0.5)	2 (0.8)	0.999
Hypertension	79 (14.3)	59 (24.9)	< 0.001
Hyperlipidemia	4 (0.7)	1 (0.4)	1.000
Atrial fibrillation	1 (0.2)	2 (0.8)	0.448

BMI, Body mass index; LDH, Lactate dehydrogenase; MPV, Mean platelet volume; n, Number; PDW, Platelet distribution width; SD; Standard deviation; VTE, Venous thromboembolism

### Univariate and multivariate analyses

The univariate regression analysis suggested that 14 risk factors were associated with a greater risk of cancer-associated thrombosis. These factors were age, tumor stage, cancer type, cerebrovascular disease, hypertension, personal history of VTE, and prechemotherapy laboratory variables, including blood leucocyte count, MPV, PDW, albumin concentration, LDH concentration, D-dimer concentration, fibrinogen concentration, blood hemoglobin concentration ≤ 100 g/dL or use of erythropoiesis-stimulating agents. In the multivariate logistic regression analysis, age ≥ 60 years, tumor stages III-IV, prechemotherapy PDW ≤ 12.6%, prechemotherapy albumin concentration ≤ 38.8 g/L, prechemotherapy LDH concentration ≥ 198 U/L, prechemotherapy D-dimer concentration ≥ 1.72 µg/mL, prechemotherapy blood hemoglobin concentration ≤ 100 g/dL or use of erythropoiesis-stimulating agents, and cancer types were independently correlated with VTE in solid tumor patients (Table 2).

**Table 2:** Multivariate analysis of cancer-associated thrombosis risk factors

Variable	Hazard ratio	95% CI	P value
Age (≥ 60 versus < 60)	2.702	1.816–4.020	< 0.001
Cancer type			0.114
Very high risk	0.274	0.042–1.788	0.176
High risk	0.325	0.109–0.973	0.045
Tumor stage (I-II versus III-IV)	2.514	1.547–4.085	< 0.001
Leucocyte count (≥ 11 × 10^9/L versus < 11 × 10^9/L)	1.528	0.769–3.038	0.226
Hemoglobin ≤ 100 g/dL or using erythropoiesis-stimulating agents (yes versus no)	2.586	1.538–4.347	< 0.001
MPV (≤ 10.7 fL versus > 10.7 fL)	0.942	0.483–1.836	0.860
PDW (≤ 12.6% versus > 12.6%)	2.387	1.207–4.719	0.012
Albumin (≤ 38.8 g/L versus > 38.8 g/L)	2.057	1.356–3.121	0.001
LDH (≥ 198 U/L versus < 198 U/L)	2.441	1.649–3.612	< 0.001
D-Dimer (≥ 1.72 µg/mL versus < 1.72 µg/mL)	4.920	3.311–7.312	< 0.001
Fibrinogen (> 4 g/L versus ≤ 4 g/L)	0.680	0.447–1.033	0.071
Personal history of VTE (with versus without)	4.309	0.356–52.175	0.251
Cerebrovascular disease (with versus without)	1.409	0.409–4.853	0.587
Hypertension (with versus without)	1.586	0.970–2.593	0.066

LDH, lactate dehydrogenase; MPV, mean platelet volume; PDW, platelet distribution width

### Nomogram construction and validation

**Fig. 2:**
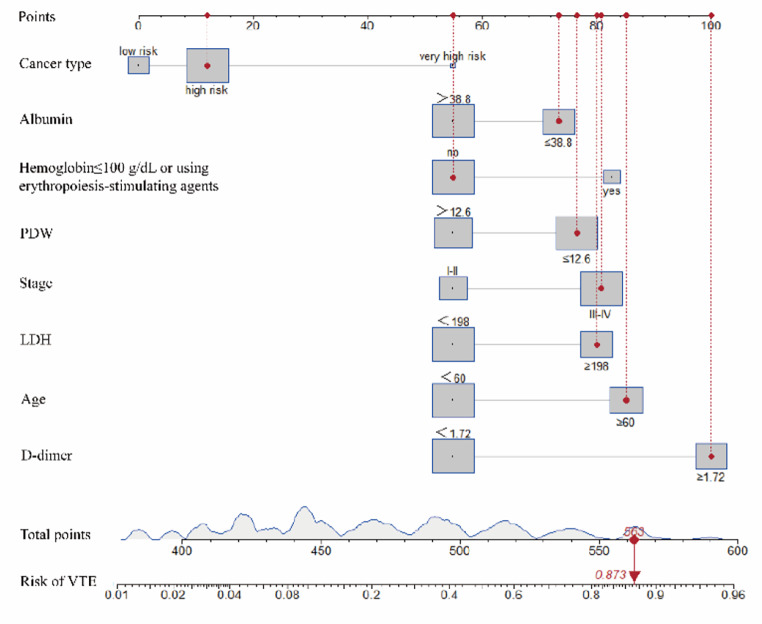
A nomogram predicting the risk of VTE for solid cancer patients. The usage of the nomogram was exampled by a patient. The patient was 62 years old and diagnosed with stage III lung cancer with no use of erythropoiesis-stimulating agents. The values of pre-chemotherapy variables of the patient were as follows: BMI 22.3 kg/m^2^, platelet count 162 × 10^9/L, leucocyte count 6.32 × 10^9/L, hemoglobin concentration 152 g/dL, platelet distribution width (PDW) 12.3%, albumin 27.9 g/L, lactate dehydrogenase (LDH) 468 U/L, and D-dimer 14.12 µg/mL. Each variable was given an associated score on the point scale axis at the top. The total score (563) was the sum of each single score and located on the Total Points axis. The risk of VTE was estimated by projecting the total score to the point scale axis at the bottom. Accordingly, the risk of VTE of this patient was 87.3%. While, based on the Khorana predictive model, the total point was 1 for high risk cancer type and the patient was divided into the intermediate risk group.

**Fig. 3:**
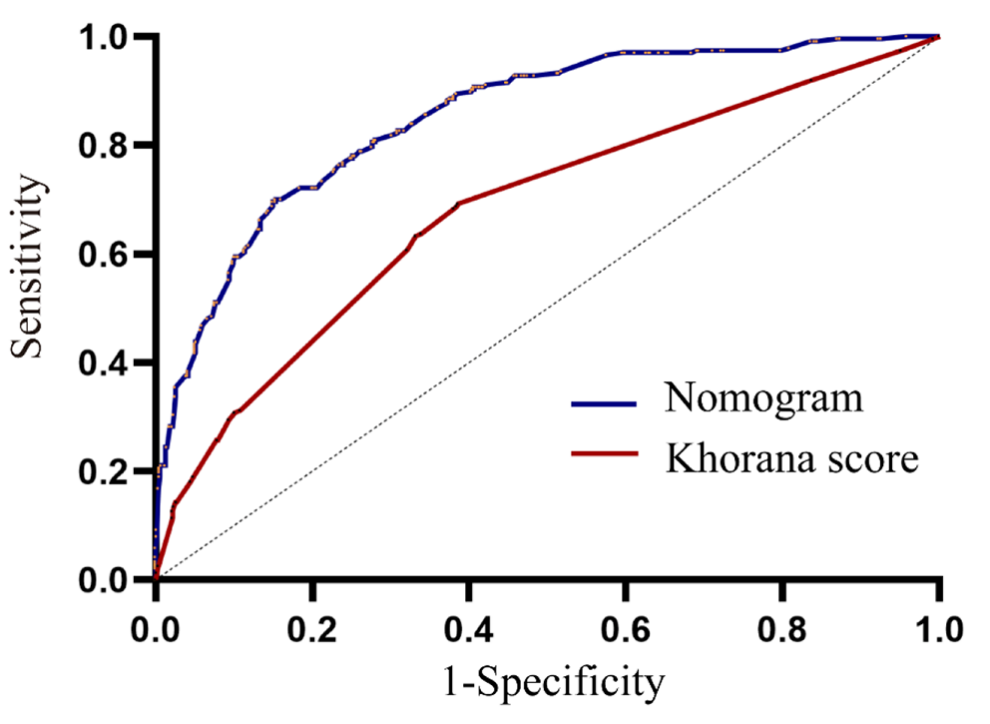
ROC curve of the nomogram and the Khorana score

**Fig. 4:**
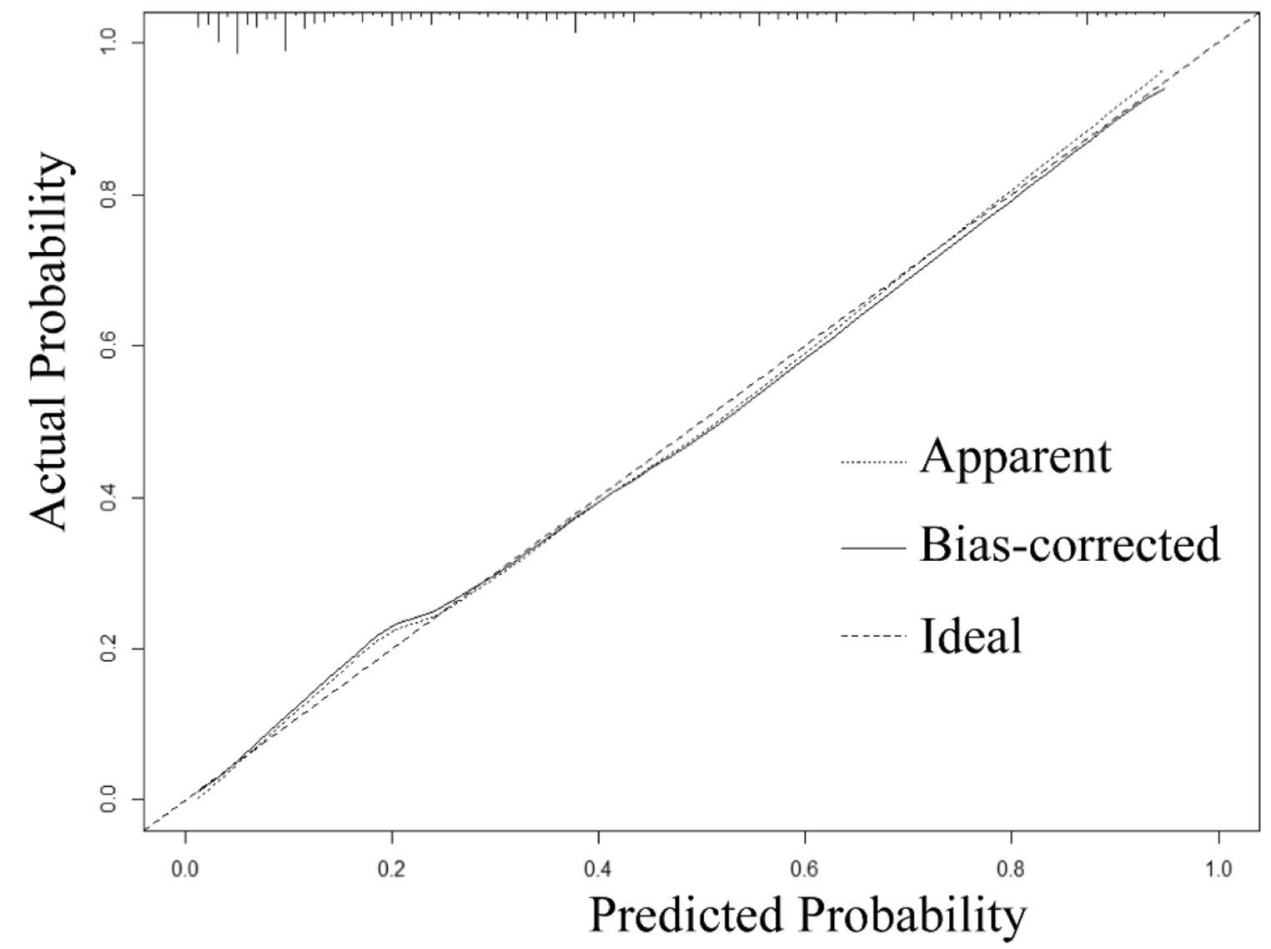
The calibration curves for the nomogram

Based on the results of the multivariate analysis, eight independent risk factors were combined into the nomogram (Fig. 2). In the nomogram, the variables were assigned scores. We were able to calculate the total score, the sum of each score, to assess the risk probability of VTE. The ROC curve and calibration plot were used to verify the performance of the nomogram (Fig. 3). The area under the ROC curve was 0.852 [95% confidence interval (CI) 0.823 to 0.880]. In addition, we used the bootstrap method to sample for internal verification of the nomogram 1000 times repeatedly, and the calculated C-index was 0.843. As the calibration curve displays in Fig. 4, there are high consistencies between the predicted and actual probabilities. In conclusion, the nomogram had excellent discrimination and calibration.

### Comparison with the khorana risk score

**Fig. 5:**
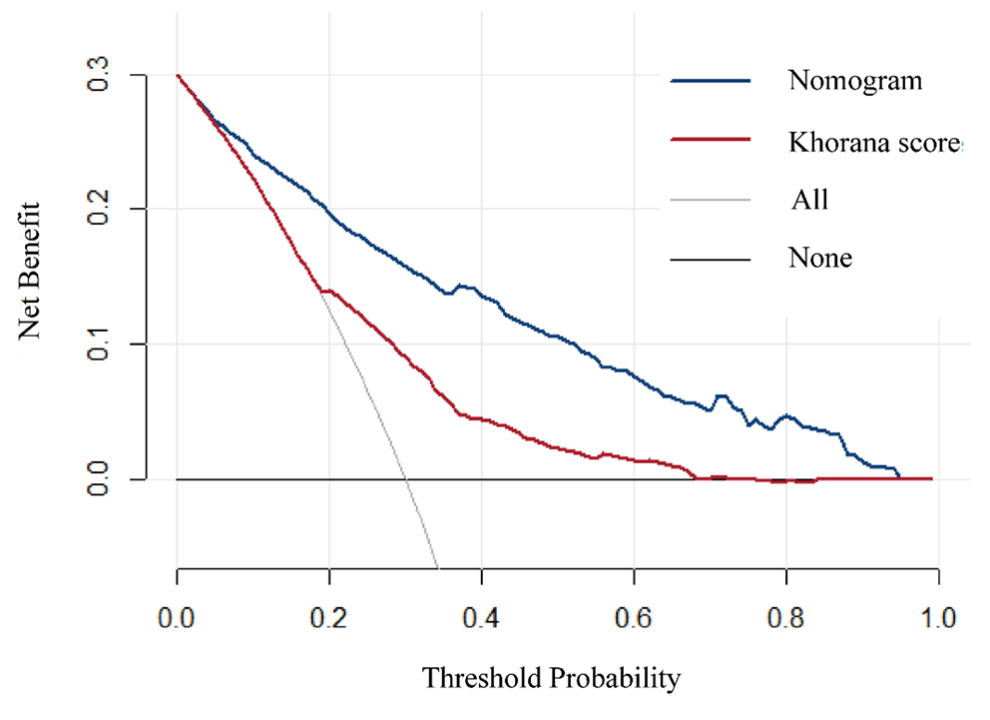
Decision curve analysis of the nomogram and the Khorana score for solid tumor patients

In this study, we found that among the five items of the Khorana score, only hemoglobin less than 100 g/L or the use of red cell growth factors and cancer types were significantly associated with VTE. Among the VTE patients, 24 were indicated as high VTE risk by the Khorana risk score, and 213 were indicated as low to moderate VTE risk. In those without VTE, 27 were indicated as having a high VTE risk, and 527 were indicated as having a low to moderate VTE risk. The ROC curve was used to verify the Khorana risk score (Fig. 3), and the C-index of the Khorana score was 0.681 (95% CI 0.639 to 0.723). IDI and DCA curves were used to compare this nomogram and the Khorana score in terms of clinical benefits. The IDI was 0.243 (95% CI 0.204 to 0.282, P < 0.05). DCA curves show that the nomogram has a better predictive ability (Fig. 5).

## Discussion

The administration of anticoagulants in cancer patients is challenging. Therefore, we developed an intuitive prediction model to identify the risk of VTE in cancer patients. We selected eight variables that were predictive of VTE and integrated them into the nomogram. Among these variables, cancer type was the most important risk factor for cancer-associated thrombosis, followed by D-dimer and age. The internal validation and decision-curve analysis demonstrated that the nomogram had great discrimination, calibration, and clinical utility.

Previous studies have proposed that cancer types are responsible for the incidence of VTE. Aggressive malignancies conferred a higher risk of thromboembolic events than indolent tumors. A cohort study using linked United Kingdom databases reported that the incidence rate of VTE in pancreatic cancer was the highest. Lung, stomach, esophagus, liver, ovary, and brain cancers induced high risks, while low risks were seen in patients with breast, oral cavity, and laryngeal cancers [2]. A population-based cohort study also indicated that pancreatic cancer induced the highest risk. Liver, ovary, stomach, esophagus, brain, colon, rectum, kidney, bladder, and uterus cancers were associated with high 6-month cumulative VTE incidence, while breast cancer induced low risk [5]. A systematic review and an epidemiologic study showed similar results, which were in accordance with our findings [17, 18]. Besides, a survey of hospitalized cancer patients indicated that high risk of venous thromboembolism was also seen in prostate cancer patients [19]. One possible explanation is that cancer types with low risks are commonly diagnosed at an earlier stage than those with great risks.

Several studies have demonstrated that D-dimer is a significant biomarker of VTE, which is consistent with our results. D-dimer is produced by the degeneration of fibrin, and when it is elevated, it is a marker of the activation of hemostasis and fibrinolysis [14]. Multiple studies have suggested that D-dimer is used to exclude thrombosis and predict VTE and its recurrence risk [20, 21]. D-dimer has been previously incorporated into the CATS score and the nomogram developed by Pabinger et al. [22, 23].

In some previous studies, serum albumin and LDH have been identified as independent risk factors for thrombosis in patients with malignancies [24–26]. In accordance, our study showed that patients with LDH levels above 198 U/L had a 2.4-fold increased risk of VTE and patients with albumin levels below 38.8 g/L had a 2.1-fold increased risk of thrombotic complications. LDH is regarded as a biomarker of tumor burden, disease activity, and tissue damage. Reduced serum albumin represents poor general health and a less favorable prognosis. A study indicated that serum albumin was negatively correlated with fibrinogen and factor VIII levels [27]. One hypothesis is that elevated LDH and reduced albumin might reflect a hyperinflammatory or hypercoagulable state [27–30]. Another potential explanation is that albumin might be a significant anticoagulant. Albumin inhibits the formation and activation of thromboxane A2, the platelet agonist, by binding arachidonic acid [31]. In addition, albumin inhibits the aggregation of platelets by binding platelet-activating factor (PAF) [32]. Besides, albumin affects the platelet aggregation inhibitors nitric oxide (NO) and prostacyclin (PGI2) [33, 34]. Albumin can induce NO production and prevent PGI2 degradation. The role of LDH and albumin in the development of thrombotic disorders warrants further investigation.

Anemia is a prevalent complication in tumor patients. Using erythropoiesis-stimulating agents as treatment for anemia is common in clinical practice. Some studies reported that using erythropoiesis-stimulating agents induced an increased risk of thrombotic events [35, 36]. Khorana et al. regarded hemoglobin reduction or the use of erythropoiesis-stimulating agents as an independent risk factor for VTE, which was consistent with our findings [9]. One potential explanation is that decreased hemoglobin concentration is correlated with low blood viscosity, which predisposes endothelial dysfunction [37, 38]. Endothelial cells in the vascular bed play a significant role in the antithrombotic mechanism. Thus, anemic patients are prone to thrombosis. The exact mechanistic pathway of hemoglobin protein in thrombosis is unclear yet. Further studies are warranted.

The present study also identified PDW as a risk factor affecting the generation of VTE. To date, only one study has suggested that PDW is an independent predictor for thrombosis events in patients with cervical carcinoma [39]. One possible explanation is that PDW is associated with platelet function. Relevant studies reported that reduced PDW values reflected the activation of platelets and that PDW was positively correlated with the coagulation times stimulated by bacterial endotoxin or tumor necrosis factor (TNF-α) [40].

Cancer patients with thrombotic complications are prone to poor outcomes. A useful risk stratification tool is warranted. Nomograms have been confirmed as a practical statistical model for quantifying risk in individual tumor patients. Therefore, we have developed an intuitive nomogram model to guide clinical treatments. Moreover, we assessed the validity of the Khorana risk score. The incidence of thrombosis was 47.0%, 36.4%, and 17.7% in the high-risk, intermediate-risk, and low-risk groups, respectively. Additionally, 10.1% of thromboembolic events occurred in patients with a high-risk Khorana score. These pieces of evidence indicate that the Khorana risk score may help identify patients who might benefit from thromboprophylaxis, but its sensitivity needs improvement. Compared with the Khorana risk score, the nomogram had a better performance.

Some encountered limitations in this study are as follows: first, this retrospective study analyzed single-institution clinical data and used bootstrapping for internal validation rather than an internal validation cohort. Although internal validation indicated that the nomogram has excellent discriminative and calibrating abilities, further external validation is needed. Secondly, considering that several prediction models, especially for hematological malignancies, have been developed, only solid tumor patients were enrolled in this study. Therefore, this model might not be applicable to hematological patients. Third, the time of cancer diagnosis, when data were collected, was heterogeneous. And due to insufficient documentation, the large and discrepant proportions of patients were excluded in this study. Finally, there may be problems with generalizability due to the use of internally derived cut-offs.

## Conclusion

In conclusion, we created and internally validated a nomogram to predict the risk of VTE in solid tumor patients. Given its performance, this nomogram could be used to select cancer patients at high risk for VTE and guide thromboprophylaxis treatment in clinical practice, provided it is validated in an external cohort.
